# Synthesis and Characterization of New Chlorhexidine-Containing Nanoparticles for Root Canal Disinfection

**DOI:** 10.3390/ma9060452

**Published:** 2016-06-07

**Authors:** Ridwan Haseeb, Michael Lau, Max Sheah, Francisco Montagner, Gina Quiram, Kelli Palmer, Mihaela C. Stefan, Danieli C. Rodrigues

**Affiliations:** 1 Department of Bioengineering, University of Texas at Dallas, 800 W Campbell, Richardson, TX 75080, USA; rbh110020@utdallas.edu (R.H.); mxl110330@utdallas.edu (M.L.); mms097020@utdallas.edu (M.S.); gjq140030@utdallas.edu (G.Q.); mihaela@utdallas.edu (M.C.S.); 2 Department of Conservative Dentistry, Dental School, Federal University of Rio Grande do Sul, Rua Ramiro Barcelos 2492, Porto Alegre–RS 90460-001, Brazil; 3 Department of Molecular and Cell Biology, University of Texas at Dallas, 800 W Campbell, Richardson, TX 75080, USA; Kelli.Palmer@utdallas.edu; 4 Department of Chemistry, University of Texas at Dallas, 800 W Campbell, Richardson, TX 75080, USA

**Keywords:** dentin tubules, dentin permeability, chlorhexidine, nanoparticles, encapsulation

## Abstract

Root canal system disinfection is limited due to anatomical complexities. Better delivery systems of antimicrobial agents are needed to ensure efficient bacteria eradication. The purpose of this study was to design chlorhexidine-containing nanoparticles that could steadily release the drug. The drug chlorhexidine was encapsulated in poly(ethylene glycol)–*block*–poly(l-lactide) (PEG–*b*–PLA) to synthesize bilayer nanoparticles. The encapsulation efficiency was determined through thermogravimetric analysis (TGA), and particle characterization was performed through microscopy studies of particle morphology and size. Their antimicrobial effect was assessed over the endodontic pathogen *Enterococcus faecalis*. The nanoparticles ranged in size from 300–500 nm, which is considered small enough for penetration inside small dentin tubules. The nanoparticles were dispersed in a hydrogel matrix carrier system composed of 1% hydroxyethyl cellulose, and this hydrogel system was observed to have enhanced bacterial inhibition over longer periods of time. Chlorhexidine-containing nanoparticles demonstrate potential as a drug carrier for root canal procedures. Their size and rate of release may allow for sustained inhibition of bacteria in the root canal system.

## 1. Introduction

The treatment of an infected root canal has been based on nonspecific elimination of intraradicular microorganisms through the application of broad-spectrum antimicrobial approaches [1]. Nevertheless, it has been shown that it is nearly impossible to obtain complete elimination of microorganisms in the root canal system [2,3]. Therefore, the continued development of treatments that can effectively penetrate dentin to eliminate root canal infection is a priority in clinical endodontic research.

Currently, the most frequent intracanal medicaments employed to treat infected root canals include calcium hydroxide (Ca(OH)_2_), potassium iodine (KI), and chlorhexidine (CHX). The efficacy of root canal disinfectants can be influenced by several factors such as pH, serum proteins, collagen, and dentin among others [4,5,6]. However, *in vitro* studies have demonstrated that CHX is more effective in eliminating bacteria from internal dentinal tubules in comparison to the other disinfectants when dispersed in liquid or hydrogel systems [7,8]. Besides its proven antimicrobial activity and non-toxicity at low dosages, CHX provides substantivity to dentin tissues, which may offer protection against microbial colonization for extended periods of time after treatment [7,9]. These attributes make CHX a potent disinfectant in root canal treatment. Dentin permeability and the complex anatomy of the root canal, however, impose challenges to the penetration and subsequent action of these disinfectants. Gomes *et al.* [10] demonstrated *in vitro* that medicaments containing 2% CHX were able to diffuse into the dentin, reaching the external root surface. The information on the length of activity time of the various agents in the root canal is limited.

Nanoparticles in recent years have been employed in several clinical applications [11]. In endodontics, nanoparticles have been suggested to act as irrigants [12], incorporated into intracanal medicaments [13] or root canal sealers [14,15]. Nanoparticle technology for drug delivery includes nanoencapsulation, which is the coating of a substance within another material, typically a polymer based system. It aims to maximize the therapeutic efficacy while minimizing undesirable side effects due to the control of the drug bioavailability and release [16,17]. There is scarce data in the current literature on the synthesis, characterization, and application of nanoencapsulated medicaments that are typically employed in root canal treatment. Shrestha and Kishen [18] evaluated the effect of rose bengal-functionalized chitosan nanoparticles associated with photodynamic therapy over monospecies bacteria/biofilms and assessed their antibiofilm efficacy on a multispecies biofilm grown on dentin. Shrestha *et al.* [19] examined the ability of the temporally controlled release of bovine serum albumin from chitosan nanoparticles to regulate the alkaline phosphatase activity in stem cells from apical papilla.

Nanoparticles produced by drug nanoencapsulation have specific characteristics such as size, release pattern, and activity, which are factors determined by the synthetic method employed, polymer system of choice, and polymer molecular weight. Therefore, protocols should be conducted to achieve proper nanoencapsulation of medicaments, taking into account the stability of the system or release profile to achieve the desirable antiseptic activity inside a specific target or tissue. Provided the anatomical complexity of the root canal, permeability of dentin, and limited penetration of medicaments in the dentin, the goal of this study is to develop and characterize a novel CHX-encapsulated system for root canal applications. In this work, the biodegradable and biocompatible block copolymer of choice was poly(ethylene glycol)–*block*–poly(l-lactide) (PEG–*b*–PLA) to create CHX loaded nanoparticles. PEG–*b*–PLA bilayer nanoparticles have advantages for drug delivery, such as small size and hydrophobic and hydrophilic functionalities in the polymer backbone that improves *in vivo* half-life. These polymeric nanoparticles were characterized for size, morphology, and drug loading proficiency. The nanoparticles were found to be small enough to penetrate dentin tubules, dispersed well in a hydrogel matrix used as a carrier system, and enhanced bacterial inhibition over longer periods of time.

## 2. Results

### 2.1. Particle Synthesis

**Figure 1:**
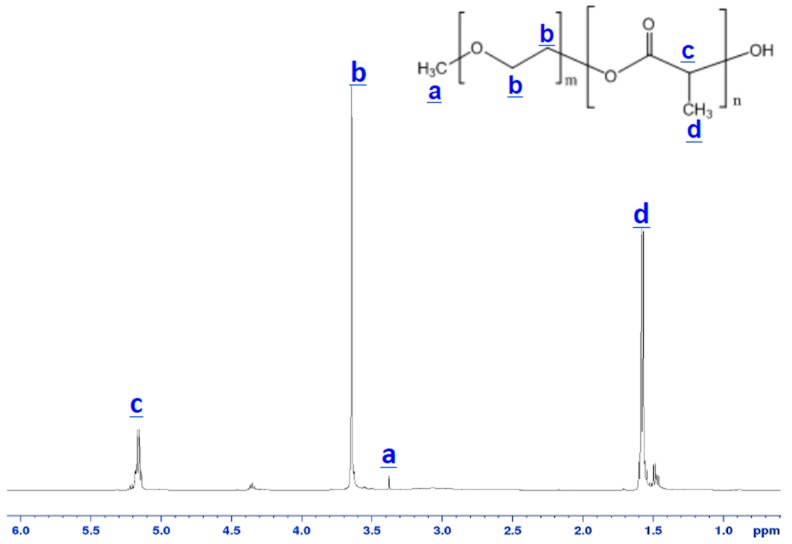
^1^H NMR spectra of the synthesized poly(ethylene glycol)–*block*–poly(l-lactide) (PEG–*b*–PLA) block copolymer.

The obtained PEG–*b*–PLA block copolymer was characterized by ^1^H NMR. Figure 1 shows the ^1^H NMR spectra of the PEG–*b*–PLA block copolymer and indicates that the block copolymer was synthesized successfully. The number-average molecular weight of the PEG–*b*–PLA block copolymer was 5756 daltons with a ratio of 2 PLA units to 1 PEG unit. The peaks at 3.64 ppm and 3.38 ppm corresponded to methylene units and CH_3_O– in the mPEG block, respectively. Signals at 1.47 ppm and 5.16 ppm could be attributed to the hydrogen atoms of CH_3_– and CH– groups for PLA segments, respectively.

### 2.2. Encapsulation Efficiency

**Figure 2:**
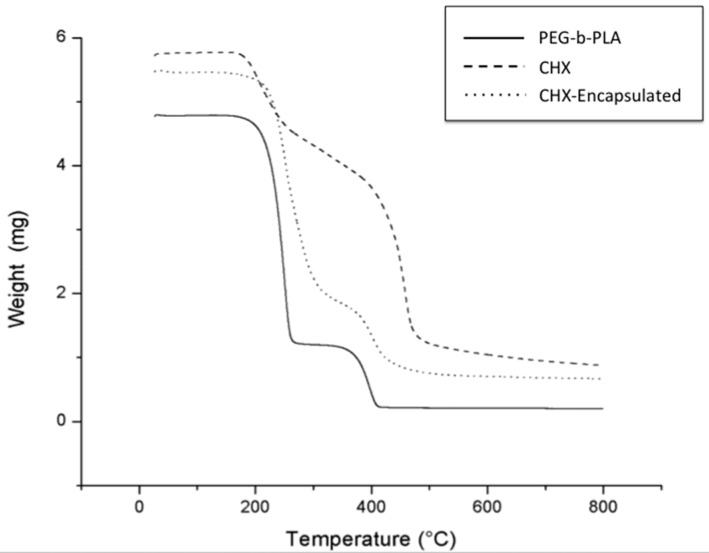
Measurements of the amount of weight change of PEG–*b*–PLA, chlorhexidine (CHX), and CHX-encapsulated. Thermogravimetric analysis (TGA) curves showing mass loss as a function of temperature. Note that thermal transitions for the CHX-encapsulated product most resemble the behavior and transitions measured for the PEG–*b*–PLA polymer due to the proportion of PEG–*b*–PLA being much higher than CHX.

The synthesis employed proved to be a simple and reproducible method to encapsulate CHX for controlled release of CHX. Investigation of the thermal behavior of synthesized products using thermogravimetric analysis (TGA) illustrated that the encapsulation of CHX was achieved. The measured mass drop of the materials as they decomposed using TGA (Figure 2) illustrates that the encapsulation process resulted in structural changes with the incorporation of CHX in PEG–*b*–PLA, as there is a slight shift in the decomposition point of the materials. TGA also provided information on the encapsulation efficiency of CHX in PEG–*b*–PLA.

**Figure 3:**
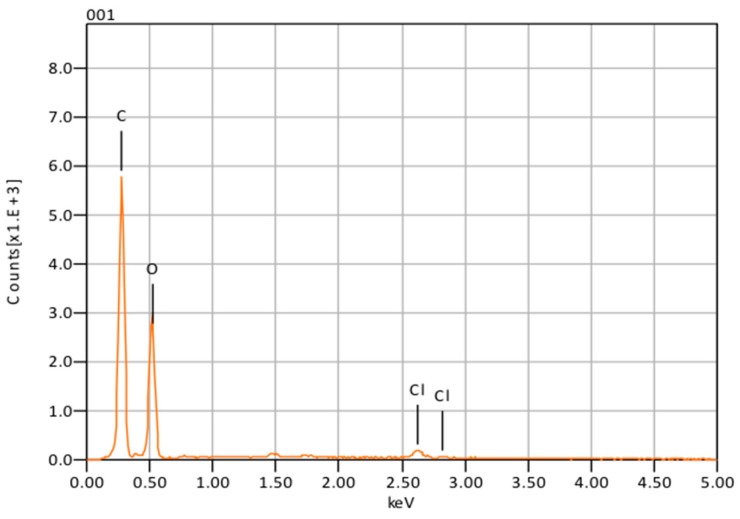
Nanoparticle composition obtained with energy dispersive X-ray spectroscopy (EDS).

The synthetic method employed allowed for an average encapsulation efficiency of 70%. Energy dispersive X-ray spectroscopy (EDS) analysis confirmed the presence of CHX in the encapsulated nanoparticles by signal emission of chlorine (Cl) at 2.7 keV (Figure 3).

### 2.3. Particle Morphology and Composition

**Figure 4:**
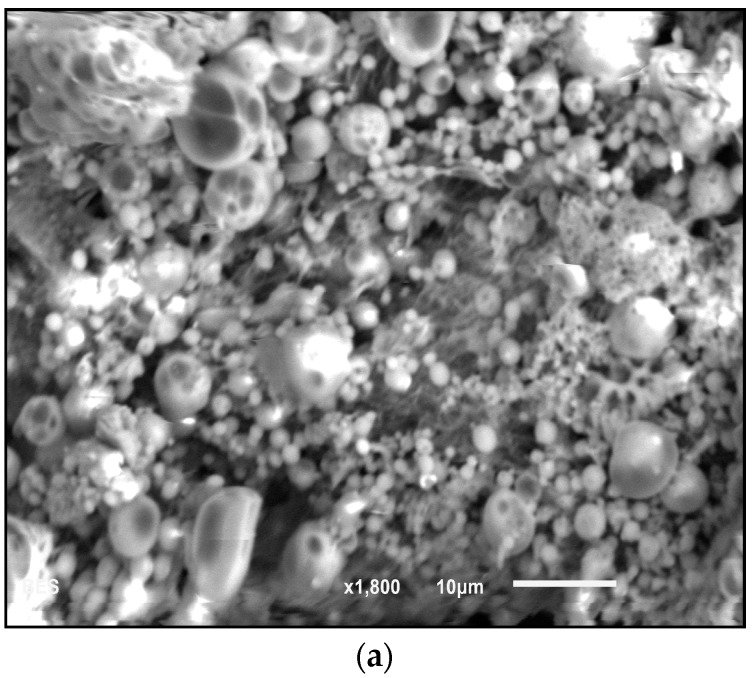

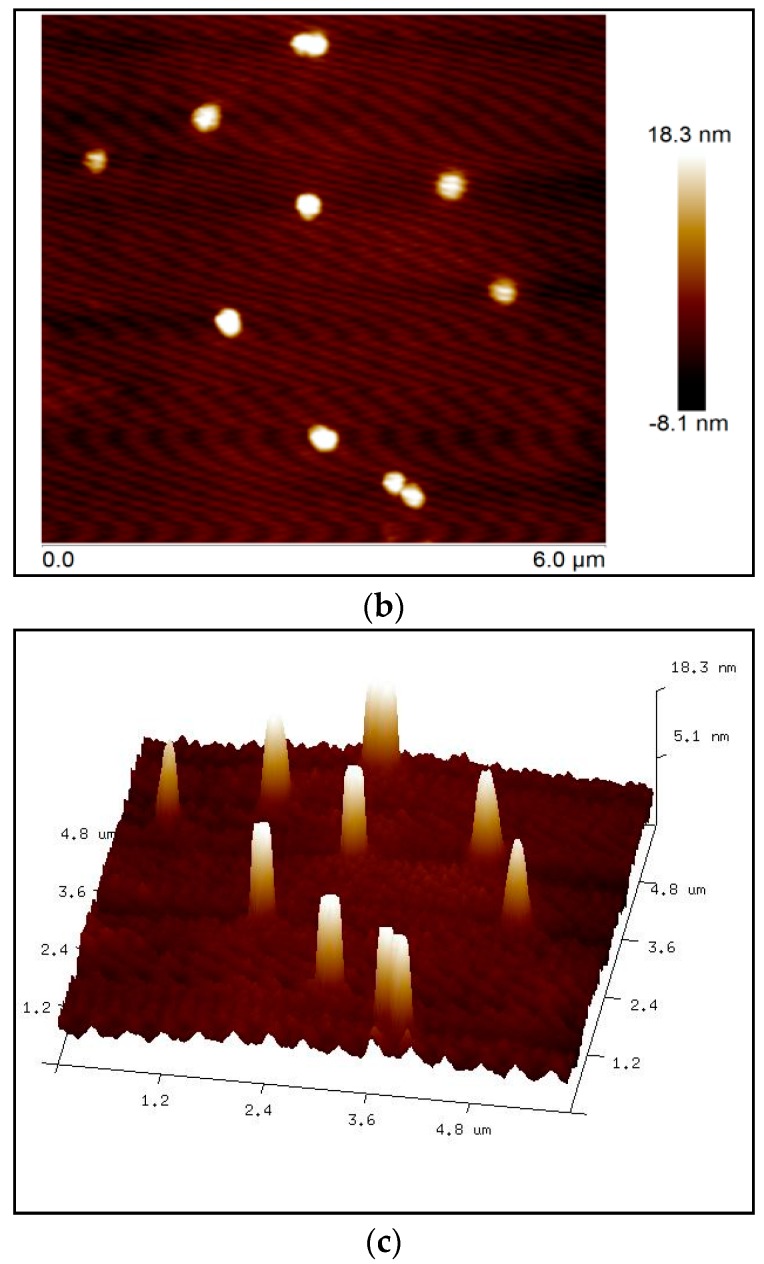
(**a**) Morphological analysis obtained with scanning electron microscopy (SEM) images of CHX-encapsulated nanoparticles. Note that clumping prevented measurement of individual nanoparticle size. Atomic force microscopy (AFM) (**b**) 2D surface topography of nanoparticles and (**c**) 3D image.

Scanning electron microscopy (SEM) at 1800× magnification (Figure 4a) was used to verify the physical characteristics of the nanoparticles and presence of clumps. The analysis showed that CHX-encapsulated formed large clumps in the dry state, which prevented accurate analysis using this technique. Atomic force microscopy (AFM) was therefore used to determine size. AFM revealed that nanoparticles individually ranged in size from 300–500 nm in diameter (Figure 4b,c). The size was confirmed by probing numerous areas on the glass slides containing the nanoparticles using topographical and 3D imaging. From dynamic light scattering (DLS) analysis, the nanoparticle’s average size was found to be 342 nm.

### 2.4. Antimicrobial Effectiveness

The ability of the CHX-encapsulated nanoparticles to retain and release CHX was investigated with zone of inhibition (ZOI) measurements. The ZOI generally became smaller as the filtered nanoparticles that spent greater time immersed in phosphate buffer saline (PBS) were placed on bacterial lawns. Control nanoparticles (synthesized without the addition of CHX) showed no ZOI, indicating that the polymer itself did not have antimicrobial activity. Table 1 shows the proportion of nanoparticle mass diameter compared to the ZOI. The table shows that nanoparticles immersed for 14 days and those immersed for 21 days displayed similar ZOIs.

**Figure 5:**
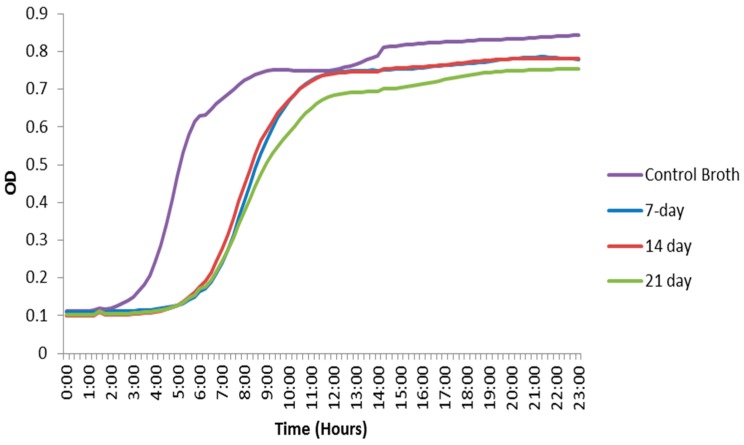
Optical density curves of *E. faecalis*. Broth that had contained CHX-encapsulated nanoparticles show a growth delay compared to the positive control broth that had not contained any nanoparticles.

The optical density (OD) data from filtered bacterial broth that had contained the nanoparticles for 7, 14 and 21 days obtained through the plate reader showed the antimicrobial effect displayed by the CHX-encapsulated nanoparticles. The onset of *E. faecalis* exponential growth was delayed by approximately three hours for nanoparticle immersion solutions as compared to the control broth, and the final OD was slightly lower in the broths that had contained the CHX nanoparticles *vs.* the control. The OD data at the intermediate time period when the control was entering the growth phase was also analyzed using a one-way ANOVA. When the growth curves of the broth that had contained the nanoparticles were compared to the control, a significant difference was observed (*P* ≤ 0.05). The results also indicated that the nanoparticle mass remained effective for the period investigated (up to 21 days). However, the final ODs as well as the lag for the growth phases remained nearly the same (Figure 5).

## 3. Discussion

The present study discussed the synthesis, characterization, and antimicrobial effectiveness of a new drug delivery system designed to allow for extended release of CHX inside the root canal system. It was hypothesized that nanoparticles prepared with an appropriate size and a controlled CHX-release profile could carry the medicament deep into dentin tubules, allowing for sustained inhibition of bacteria in the root canal system.

Chlorhexidine has been used previously in hydrogel form and in liquid formulations in root canal treatment. CHX is well-known to be rapidly released from poly(lactic acid) microparticles [20]. Therefore, in order to enhance bacterial elimination from infected root canal systems and to create an environment that is ideal for periapical healing, it is highly desirable to extend the release period of CHX *in situ* beyond the delivery systems currently in use. Nanoencapsulation can provide better control while further extending the release period of the medicament in dentin tissues. The antibacterial activity of CHX in different concentrations and preparation forms has been extensively tested. In endodontics, CHX has been employed as an intracanal medicament in a 2% concentration, alone or associated with calcium hydroxide [21]. It should also be emphasized that, the higher the drug concentration, the higher its side effects.

The bacterial activity of CHX loaded poly(ε-caprolactone) nanocapsules and CHX digluconate against *S. epidermis* was studied in [22]. The CHX carrier system was observed to improve drug targeting of bacteria, further reducing bacterial growth onto skin in relation to CHX digluconate. In the present study, CHX was encapsulated in a poly(ethylene glycol)–*block*–poly(l-lactide) (“PEG–*b*–PLA”). The choice of PEG–*b*–PLA is optimal as poly(l-lactic acid) has long been known for its biocompatibility and biodegradation properties [23]. It is a common choice to safely encapsulate bioactive drugs and control their release [24]. PEG has been copolymerized with PLA to create a block polymer and facilitate drug release due to its dual nature, hydrophilic-hydrophobic, which can increase pore formation in the nanoparticles along with a rise in the rate of polymer degradation. PLA microspheres degrade through a hydrolytic chain cleavage reaction affecting both the surface and the bulk properties of spheres while exhibiting no serious health risks [24]. All these properties make the copolymer a viable candidate to both encapsulate and release CHX. The PEG–*b*–PLA nanoparticles were prepared through the oil-in-water emulsion technique. The goal of this methodology was to synthesize nanoparticles smaller than 1 µm in order to facilitate penetration of the nanoparticles deep into the tubules. The encapsulation process resulted in nanoparticles with a size ranging much smaller than the diameter of dentin tubules [25]. Atomic force microscopy revealed that the size of the nanoparticles ranged from 300 to 500 nm (Figure 4), which was verified by dynamic light scattering (DLS).

The PEG–*b*–PLA nanoparticles prepared through the oil-in-water emulsion technique resulted in high material yields and drug encapsulation efficiencies as high as 80%. TGA revealed that CHX was incorporated into the polymer as demonstrated by weight changes of decomposition reactions of the CHX-containing nanoparticles in comparison to starting materials—CHX and the PEG–*b*–PLA block copolymer (Figure 2).

The presence of chlorine peaks (Figure 3) in the EDS spectra are evidence of CHX-encapsulated nanoparticles. As this element is characteristic of CHX and not the polymer, there is evidence to suggest that the CHX is being absorbed on the surface of the nanoparticles and encapsulated. Although SEM showed that the nanoparticles can form large clumps during the lyophilization process, these were significantly reduced when the nanoparticles were dispersed in a hydrogel matrix made out of 1% Natrosol™ (hydroxyethyl cellulose) in water.

Nanoparticle disperssion within the hydrogel matrix consists of polymer chains from the hydrogel forming weak bonds with many nanoparticles. This in turn produces a loosely interlinked network of polymers and nanoparticles [26]. Since each connection point is rather weak, the bonds breakdown under mechanical stress, for instance, when injected through syringes [27]. When the shear forces subside, the polymers and nanoparticles form new connections with the walls of dentinal tubules and within the hydrogel itself. Now, the high water content and large pore sizes of this hydrogel will be sufficient to trigger hydrolysis of the backbone ester groups in PLA and the oxidation of the ether backbone in PEG, thereupon diffusing the chlorhexidine.

Microorganisms may penetrate inside dentin to different extents and may survive inside the tubules, even after the use of the currently employed disinfection protocols. In a clinical study, Siqueira *et al.* [28] demonstrated that chemomechanical preparations with 2.5% NaOCl as irrigant significantly reduced the number of bacteria in the canal, but allowed for microbial recovery through cultivation in more than one-half of all cases. Authors also reported that a seven-day intracanal dressing with Ca(OH)_2_/camphorated paramonochlorophenol (CPMC) paste significantly increased the number of culture-negative cases. However, positive cultures were still obtained. Furthermore, *Enterococcus faecalis*, a facultative anaerobe that can be isolated from persistent/secondary root canal infections, has demonstrated to be resistant to calcium hydroxide, especially when in biofilms. They can adapt to harsh environmental changes and can colonize dentinal tubules where they remain protected from medicaments. Upadya *et al.* [29] showed that *E. faecalis* biofilms were considerably more resistant to Ca(OH)_2_ solutions than free-floating cells. A fraction from the biofilm cells persisted viable even after 24-h exposure to a saturated Ca(OH)_2_ solution. It was pointed out that the increased resistance might be attributed to the biofilm structure or extrapolimeric substance. Therefore, other antimicrobial agents, with different mechanisms of action should be employed to enhance the root canal system disinfection, especially in areas that are difficult to reach with conventional instrumentation and currently employed intracanal medicament. Chlorhexidine digluconate is water-soluble and readily dissociates at physiologic pH releasing the positively charged chlorhexidine component. The bactericidal effect of the drug is due to the cationic molecule binding to extra microbial complexes and negatively charged microbial cell walls, thereby altering the cells’ osmotic equilibrium [30,31]. Chlorhexidine with a concentration of 2%, in both liquid or gel presentations, has been employed as an auxiliary chemical substance during root canal preparation or as a final irrigant [32,33,34]. Several *in vitro* studies assessed the properties of CHX as an intracanal medicament [10,35,36]. However, there are few clinical studies that employed CHX hydrogel as an intracanal medicament. Gama *et al.* [37] evaluated the incidence of postoperative pain after intracanal dressings with either 0.12% chlorhexidine digluconate gel or a calcium hydroxide/camphorated paramonochlorophenol/glycerin paste. Therefore, this study intended to develop a nanoparticle–hydrogel matrix system to be employed as an intracanal medicament that would carry CHX, allowing for its continuous release inside the root canal system.

Few studies have assessed the antimicrobial effect of antimicrobial-containing nanoparticles over endodontic pathogens, especially *Enterococcus faecalis*. The effect of rose bengal-functionalized chitosan nanoparticles over *E. faecalis* cells and multispecies biofilm structures was assessed by Shrestha and Kishen [18]. Despite the quantification of the CHX release from nanoparticles assessed through analytical methods [38,39,40], no study has evaluated the residual antimicrobial effect produced by suspended nanoparticles over time, especially against *E. faecalis* cells. In the present study, *in vitro* studies in *E. faecalis* were performed to investigate bacterial growth inhibition in the presence of CHX-encapsulated nanoparticles. The experiment was performed at different time points to investigate the length of antibacterial activity and effectiveness of the synthesized delivery system. The initial goal was to maintain CHX release for a minimum period of 7 to 14 days, which corresponds to the period that an intracanal medicament (such as calcium hydroxide paste) is often kept between appointments. The synthesized nanoparticles were immersed in both PBS and BHI (brain–heart infusion) broth. Nanoparticles removed from the PBS and placed on bacterial lawns showed a zone of inhibition after being immersed for as long as 21 days. These zones of inhibition demonstrate that CHX both elutes into the solution and remains in the particles after eluting for up to three weeks. They also provide evidence of the area where bacteria will not come into contact with the material even after eluting the drug into a solution. The broth containing the nanoparticles demonstrated a lag in the growth phase of *E. faecalis* bacteria as well as a decrease in total cell density during this lag phase, indicating inhibition and demonstrating that CHX was indeed diffusing from the nanoparticles. The diffusion of CHX is directed by chemical potential gradients arising by osmotic pressure. In addition to diffusion, CHX could be released by erosion of the polymer matrix, which leads to pore formation [41].

The initial burst release behavior of the nanoparticles could be attributed to the CHX absorbed on the surface of the nanoparticles, with subsequent releases corresponding to the encapsulated portion. Both results (ZOI and OD) suggest that the nanoparticles demonstrate a bacteriostatic effect for at least three weeks. The zone of inhibition tests showed that CHX was eluting from the hydrophobic core of the PEG–*b*–PLA nanoparticles into the PBS solution, while some CHX still remained in the nanoparticles and were still capable of antimicrobial action. Furthermore, the bacterial growth curves using the OD data demonstrated that eluting CHX into bacterial broth did have an effect on the *E. faecalis* bacteria’s growth curve. However, the size of the zones of inhibition decreased over time. Eventually, the release of CHX reached equilibrium, and the nanoparticles that had been immersed for 14 days in PBS showed similar-sized zones as those immersed for 21 days. This suggests that hydrolysis or enzymatic cleavage of the PEG–*b*–PLA backbone reached the hydrophobic core of the nanoparticle, which causes bulk erosion, hence releasing the remaining CHX during the time period between 14 and 21 days. Additionally, the bacterial growth curves in the broth eventually reached their growth phase after the initial lag and had a final OD similar to the control, albeit slightly lower.

The intent of this nanoparticle-hydrogel matrix system was to develop an intracanal medicament that allows for the distribution of the nanoparticles through the lateral tubules and accessory canals that are exposed during the root canal procedure. The nanoparticle–hydrogel matrix system should not remain in the root canal itself after the endodontist does adequate irrigations prior to canal filling. There have been studies on hydrogel formulations and their ability to insert into narrow and convoluted locations, such as in the inter-tubular dentin matrix [13,42,43]. Thus, we are using this adhesive property of hydrogels in order to deposit and fasten the nanoparticles to the walls of dentinal tubules and accessory canals.

Control PEG–*b*–PLA nanoparticles (synthesized without the addition of CHX) showed no ZOI, indicating that no reactive oxygen species (ROS) were introduced during the synthesis of the nanoparticles. ROS are partially reduced metabolites of oxygen, including hydrogen peroxide and hydroxyl radical, which can result from polymer degradation [44]; this in turn causes additional oxidative stress in many pathological pathways making them toxic to cells [45]. Free radical generation in the cell culture containing the nanoparticles can also arise from the cellular uptake of low molecular weight polymer chains that result from their degradation [46]. This likewise leads to cytotoxicity due to the stimulation of ROS and/or the accumulation of polymer degradation products inside the cell. During the 21-day antimicrobial effectiveness study of PEG–*b*–PLA nanoparticles synthesized without the addition of CHX, no inhibitory diameter was detected, indicating that, even if the polymer is degrading, cells are not being affected by its degradation products or triggering ROS production inside cells. The results from the control in this study are of significant importance in the design of nano-carrier systems for endodontic drug delivery, thus validating the well-known biodegradability and biocompatibility of nanoparticles made out of PEG–*b*–PLA and making this a safe and suitable system of CHX delivery inside dentinal tubules.

The physiochemical properties of the PEG–*b*–PLA nanoparticles will need to be further tuned in order to enhance bacterial inhibition over longer periods of time. This will allow for prolonged release in the dentin tubules to better ensure the success of a root canal procedure. Future studies will include increasing the concentration of nanoparticles in medium and assessing the nanoparticle hydrogel matrix delivery network inside the root canal system.

## 4. Materials and Methods

### 4.1. Materials

Poly(ethylene glycol) methyl ether [average molecular weight (Mn) = 2000 g/mol], l-lactide, toluene, tin (II) 2-ethylhexanoate, chlorhexidine, poly(vinyl alcohol) (PVA) [87%–90% hydrolyzed, average Mw = 30,000–70,000 g/mol], dichloromethane (DCM), 1x phosphate buffer saline (PBS), and pentane were used as received without further purification. All chemicals were purchased from Sigma-Aldrich (St. Louis, MO, USA).

### 4.2. Nanoparticles Synthesis

The process of preparing bilayer nanoparticles comprised two steps: (1) polymer synthesis followed by (2) encapsulation of the drug.

#### 4.2.1. Polymer Synthesis

Poly(ethylene glycol)–*block*–poly(l-lactide) (PEG–*b*–PLA) was synthesized in house by introducing poly(ethylene glycol) methyl ether (Mn = 2000) and l-lactide (Lactide-(3*S*)-*cis*-3,6-Dimethyl-1,4-dioxane-2,5-diene) into toluene distilled over sodium benzyl phenol at 25 °C. The mixture was subsequently placed under vacuum to remove moisture. Tin (II) 2-ethylhexanoate was added as a catalyst during continuous stirring at 100 °C for 5 h. The resulting polymer was precipitated in pentane and air-dried at room temperature overnight. The chemicals used were purchased from Sigma-Aldrich (St. Louis, MO, USA). The number-average molecular weight of the block copolymer was determined by ^1^H NMR (ADVANCE III 500 MHz, Bruker, Santa Barbara, CA, USA). Samples temperature was regulated for all measurements and was set at 25 °C.

#### 4.2.2. Encapsulation Process

An oil-water-emulsion-evaporation method was carried out for encapsulation of chlorhexidine (CHX). The oil/organic phase consisted of PEG–*b*–PLA and CHX in a 5:1 ratio, respectively, dissolved in dichloromethane (DCM). The water phase was a 1% *w*/*v* solution of poly(vinyl alcohol) and deionized water. The organic and water phases were combined and emulsified using ultra-sonication for 1 min (Branson Ultrasonics Corporation, Bransonic CPX3800H, Danbury, CT, USA). The resulting emulsion was stirred for 2 h at 25 °C at atmospheric pressure to allow the organic solvent to evaporate. Finally, the particles were centrifuged, washed, and freeze-dried.

### 4.3. Characterization

The starting and final products were characterized using multiple techniques: (1) encapsulation efficiency and (2) microscopy studies of nanoparticle morphology and size.

### 4.4. Encapsulation Efficiency

Encapsulation efficiency was found using quantitative measurement of mass change from dehydration, decomposition, and oxidation with time and temperature of the initial polymer (PEG–*b*–PLA), CHX, and CHX-encapsulated nanoparticles. Mass changes, from their physiochemical reactions, were measured using thermogravimetric analysis (TGA, Metler Toledo TGA1, Greifensee, Switzerland). This instrument detects changes in weight that occur in a material with increasing temperature (25 °C to 800 °C). A heating rate of 5 °C per minute was applied with about 5 mg of sample used for each run. The temperatures at which the polymers and CHX characteristic mass dropped were recorded and compared. For the CHX curve, the temperature at which a significant mass loss percentage occurred was recorded (CHX_Temp_) and used as a baseline temperature. As the percentage loss of CHX at this temperature was determined earlier, the percentage of CHX lost in the nanoparticles could be found as well. This percentage was used to find the amount of CHX in the encapsulated nanoparticles and subsequently used to find encapsulated efficiency of the polymer (Equation (1)). The polymer curve was used to ensure that the polymer mass loss temperature did not overlap with CHX_Temp_. CHX was detected when characteristic changes in the thermal decomposition temperatures were observed for the CHX-encapsulated polymer in comparison to the starting materials (pure PEG–*b*–PLA and pure CHX).

(1) $$Encapsulation\;Efficiency=\frac{Mass\;of\;CHX\;in\;Particles}{Mass\;of\;CHX\;used\;in\;Synthesis}\;.$$

### 4.5. Particle Morphology and Composition

The morphology and size of polymer and encapsulated nanoparticles were observed with scanning electron microscopy (SEM, JEOL JSM-6010LA, Peabody, MA, USA) and atomic force microscopy (AFM, Bruker, Bioscope Catalyst, Santa Barbara, CA, USA). For the SEM analysis, a thin layer of nanoparticles was deposited on a metallic stub. The composition of the materials was measured with energy dispersive X-ray spectroscopy (EDS, JEOL JSM-6010LA, Peabody, MA, USA), which enabled detection of the individual elements of the nanoparticles. For AFM analysis, CHX-encapsulated nanoparticles were dispersed in a hydrogel solution (1% Natrosol™ hydroxyethyl cellulose in water) and sonicated for about 30 h (Branson Ultrasonics Corporation, Bransonic CPX3800H, Danbury, CT, USA) to disrupt clumps. A drop of solution containing nanoparticles was deposited on a glass slide and was allowed to dry forming a thin film. The AFM analysis was performed by using the quantitative nanomechanics method (QNM) for compositional mapping, which, besides determining morphological features, enables quantitative measurement of nanoscale material properties. The nanoparticle’s size and size distribution were also investigated by dynamic light scattering (DLS) technique, using a non-invasive backscatter optics (NIBS) (Zetasizer Nano ZS, Malvern, Worcestershire, UK), after suspending 2 mg of the nanoparticles in 10 mL of deionized water.

### 4.6. Antimicrobial Effectiveness

The antimicrobial effect of the encapsulated nanoparticles was tested against *Enterococcus faecalis* OG1RF. CHX-encapsulated nanoparticles were immersed in phosphate buffer saline (PBS) and agitated for set time periods (1 h, 7 days, 14 days and 21 days). Then, the nanoparticles were filtered from the solution using vacuum filtration and air-dried for approximately 24 h. *E. faecalis* overnight broth culture was spread onto BHI (brain-heart infusion) agar plates and ~1 mg of dried CHX-encapsulated nanoparticles filtered previously from the PBS were placed onto the bacterial lawns in a roughly circular formation to test for zones of growth inhibition (ZOI) around the nanoparticles. The bacterial lawn plates were incubated for 24 h at 37 °C, and the ZOIs were observed. These experiments were performed with nanoparticles that were immersed in a PBS solution for 1 h, 7 days, 14 days and 21 days to investigate the potency of bacterial inhibition and whether any drug burst release was present. Digital calipers, which are measurement tools, were used to determine the diameter of the ZOI and the diameter of a batch of nanoparticles placed on the plates. As the mass, shape, and area of nanoparticles placed on the plate were variable (nanoparticles charge made placement difficult on the plate surface), the relative diameter of an assembled group of nanoparticles was measured and compared to the diameter of the ZOI. Therefore, if the ratio increased, that meant the ZOI decreased, as this would signify that the zone was closer to the nanoparticle mass. Three trials of this test were performed.

CHX-encapsulated nanoparticles were also immersed in BHI broth for varied periods (7 days, 14 days and 21 days). These time periods represent how long the nanoparticles would be active inside dentinal tubules. The nanoparticles were filtered out, and the remaining broth was kept. The broth was then inoculated with *E. faecalis* at an initial optical density at 600 nm (OD_600_) of ~0.001. Afterwards, 200 µL of the inoculated broth was aliquoted in triplicate into a 96-well plate and incubated at 37 °C for 24 h. The OD_600_ was monitored with a Monochromater-based Multi-Mode Microplate Reader (Synergy Mx, Winooksi, VT, USA) every 15 min during the 24-h incubation period. The OD data was used to generate bacterial growth curves made by averaging triplicates from three trials together, not including clear outliers obtained during the experiments. The mean values of intermediate OD readings when the control broth was entering the growth phase were analyzed statistically with one-way ANOVA (analysis of variance) method at a 5% significance level. The OD readings of the broth that had contained the CHX nanoparticles were compared to the control to verify that the broth with CHX nanoparticles was indeed causing a delay in the growth phase.

## 5. Conclusions

In this study, PEG–*b*–PLA bilayer nanoparticles for CHX delivery were successfully assembled to improve drug bioavailability and target drug delivery to dentin tubules. This synthesis allowed for the sustained inhibition of bacteria that could potentially be used in root canal systems. The bilayer polymeric nanoparticles employed featured a hydrophobic interior space that easily encapsulated CHX, a hydrophobic drug. CHX release was effective for up to 21 days with an initial burst, which may be attributed to the CHX absorbed on the surface of the nanoparticles and subsequently predominantly controlled by diffusion and degradation mechanisms. These results have potential implications for the design of CHX polymeric nanoparticles for the *in situ* treatments of the root canal.

## Figures and Tables

**Table 1:** Zone of inhibitions measured for CHX-encapsulated nanoparticles filtered from phosphate buffer saline (PBS). The values indicate the results obtained in three trials averaged together.

Days Particles Were Immersed in PBS	Ratio of Assembled Nanoparticles Diameter *vs.* Zone Diameter
1 h	0.436 ± 0.042
7	0.584 ± 0.019
14	0.659 ± 0.042
21	0.675 ± 0.043

## Abbreviations

The following abbreviations are used in this manuscript:

CHX	chlorhexidine
EDS	energy dispersive X-ray spectroscopy
^1^H NMR	proton nuclear magnetic resonance
AFM	atomic force microscopy
SEM	scanning electron microscopy
ZOI	zone of inhibition
OD	optical density

## References

[1] Siqueira J.F., Rôças I.N. (2009). Community as the unit of pathogenicity: An emerging concept as to the microbial pathogenesis of apical periodontitis. Oral Surg. Oral Med. Oral Pathol. Oral Radiol. Endod..

[2] Peters L.B., Wesselink P.R. (2002). Periapical healing of endodontically treated teeth in one and two visits obturated in the presence or absence of detectable microorganisms. Int. Endod. J..

[3] Vera J., Siqueira J.F., Ricucci D., Loghin S., Fernández N., Flores B., Cruz A.G. (2012). One-*versus* two-visit endodontic treatment of teeth with apical periodontitis: A histobacteriologic study. J. Endod..

[4] Agrafioti A., Tzimpoulas N.E., Kontakiotis E.G. (2013). Influence of dentin from the root canal walls and the pulp chamber floor on the pH of intracanal medicaments. J. Endod..

[5] Haapasalo H.K., Sirén E.K., Waltimo T.M., Ørstavik D., Haapasalo M.P. (2000). Inactivation of local root canal medicaments by dentine: An *in vitro* study. Int. Endod. J..

[6] Haapasalo M., Qian W., Portenier I., Waltimo T. (2007). Effects of dentin on the antimicrobial properties of endodontic medicaments. J. Endod..

[7] Dametto F.R., Ferraz C.C.R., Gomes B.P.F.G., Zaia A.A., Teixeira F.B., Souza-Filho F.J. (2005). *In vitro* assessment of the immediate and prolonged antimicrobial action of chlorhexidine gel as an endodontic irrigant against *Enterococcus faecalis*. Oral. Surg. Oral. Med. Oral. Pathol. Oral. Radiol. Endod..

[8] Ercan E., Ozekinci T., Atakul F., Gul K. (2004). Antibacterial activity of 2% chlorhexodine gluconate and 5.25% sodium hypochlorite in infected root canal: An *in vivo* study. J. Endod..

[9] Leonardo M.R., Tanomaru F.M., Silva L.A., Nelson F.P., Bonifacio K.C., Ito I.Y. (1999). *In vivo* antimicrobial activity of 2% chlorhexidine used as a root canal irrigation solution. J. Endod..

[10] Gomes B.P.F.A., Montagner F., Berber V.B., Zaia A.A., Ferraz C.C.R., de Almeida J.F.A., Souza-Filho F.J. (2009). Antimicrobial action of intracanal medicaments on the external root surface. J. Dent..

[11] Murthy S.K. (2007). Nanoparticles in modern medicine: State of the art and future challenges. Int. J. Nanomed..

[12] Abbaszadegan A., Nabavizadeh M., Gholami A., Aleyasin Z.S., Dorostkar S., Saliminasab M., Ghasemi Y., Hemmateenejad B., Sharghi H. (2015). Positively charged imidazolium-based ionic liquid-protected silver nanoparticles: A promising disinfectant in root canal treatment. Int. Endod. J..

[13] Javidi M., Afkhami F., Zarei M., Ghazvini K., Rajabi O. (2014). Efficacy of a combined nanoparticulate/calcium hydroxide root canal medication on elimination of *Enterococcus faecalis*. Aust. Endod. J..

[14] Barros J., Silva M.G., Rôças I.N., Gonçalves L.S., Alves F.F., Lopes M.A., Pina-Vaz I., Siqueira J.F. (2014). Antibiofilm effects of endodontic sealers containing quaternary ammonium polyethylenimine nanoparticles. J. Endod..

[15] Kesler Shvero D., Abramovitz I., Zaltsman N., Perez Davidi M., Weiss E.I., Beyth N. (2013). Towards antibacterial endodontic sealers using quaternary ammonium nanoparticles. Int. Endod. J..

[16] Álvarez A.L., Espinar F.O., Méndez J.B. (2011). The application of microencapsulation techniques in the treatment of endodontic and periodontal diseases. Pharmaceutics.

[17] Suri S.S., Fenniri H., Singh B. (2007). Nanotechnology-based drug delivery systems. J. Occup. Med. Toxicol..

[18] Shrestha A., Kishen A. (2014). Antibiofilm efficacy of photosensitizer-functionalized bioactive nanoparticles on multispecies biofilm. J. Endod..

[19] Shrestha S., Diogenes A., Kishen A. (2014). Temporal-controlled release of bovine serum albumin from chitosan nanoparticles: Effect on the regulation of alkaline phosphatase activity in stem cells from apical papilla. J. Endod..

[20] Fay F., Linossier I., Legendre G., Vallée-Réhel K. (2008). Micro-encapsulation and antifouling coatings: Development of poly(lactic acid) microspheres containing bioactive molecules. Macromol. Symp..

[21] Gomes B.P., Vianna M.E., Zaia A.A., Almeida J.F., Souza-Filho F.J., Ferraz C.C. (2013). Chlorhexidine in endodontics. Braz. Dent. J..

[22] Lboutounne H., Chaulet J.F., Ploton C., Falson F., Pirot F. (2002). Sustained ex vivo skin antiseptic activity of chlorhexidine in poly(ε-caprolacton) nanocapsule encapsulated form and as digluconate. J. Control. Release.

[23] Athanasiou K.A., Niederauer G.G., Agrawal C.M. (1996). Sterilization, toxicity, biocompatibility and clinical applications of polylactic acid/polyglycolic acid copolymers. Biomaterials.

[24] Shive M.S., Anderson J.M. (1997). Biodegradation and biocompatibility of PLA and PLGA microspheres. Adv. Drug Deliv. Rev..

[25] Lopes M.B., Sinhoreti M.A., Gonini Júnior A., Consani S., Mccabe J.F. (2009). Comparative study of tubular diameter and quantity for human and bovine dentin at different depths. Braz. Dent. J..

[26] Appel E.A., del Barrio J., Loh X.J., Scherman O.A. (2012). Supramolecular polymeric hydrogels. Chem. Soc. Rev..

[27] Salem A.K., Rose F.R., Oreffo R.O., Yang X., Davies M.C., Mitchell J.R., Shakesheff K.M. (2003). Porous Polymer and Cell Composites That Self-Assemble *in Situ*. Adv. Mater..

[28] Siqueira J.F., Magalhães K.M., Rôças I.N. (2007). Bacterial reduction in infected root canals treated with 2.5% NaOCl as an irrigant and calcium hydroxide/camphorated paramonochlorophenol paste as an intracanal dressing. J. Endod..

[29] Upadya M., Shrestha A., Kishen A. (2011). Role of efflux pump inhibitors on the antibiofilm efficacy of calcium hydroxide, chitosan nanoparticles, and light-activated disinfection. J. Endod..

[30] Greenstein G., Berman C., Jaffin R. (1986). Chlorhexidine: An adjunct to periodontal therapy. J. Periodontal..

[31] Hugo W.B., Longworth A.R. (1964). Some aspects of the mode of action of chlorhexidine. J. Pharm. Pharmacol..

[32] Böttcher D.E., Sehnem N.T., Montagner F., Parolo C.C.F., Grecca F.S. (2015). Evaluation of the effect of *Enterococcus faecalis* biofilm on the 2% chlorhexidine substantivity: An *in vitro* study. J. Endod..

[33] Gomes B.P., Martinho F.C., Vianna M.E. (2009). Comparison of 2.5% sodium hypochlorite and 2% chlorhexidine gel on oral bacterial lipopolysaccharide reduction from primarily infected root canals. J. Endod..

[34] Rôças I.N., Provenzano J.C., Neves M.A., Siqueira J.F. (2016). Disinfecting Effects of Rotary Instrumentation with Either 2.5% Sodium Hypochlorite or 2% Chlorhexidine as the Main Irrigant: A Randomized Clinical Study. J. Endod..

[35] Almyroudi A., Mackenzie D., McHugh S., Saunders W.P. (2002). The effectiveness of various disinfectants used as endodontic intracanal medications: An *in vitro* study. J. Endod..

[36] Gomes B.P., Vianna M.E., Sena N.T., Zaia A.A., Ferraz C.C., de Souza Filho F.J. (2006). *In vitro* evaluation of the antimicrobial activity of calcium hydroxide combined with chlorhexidine gel used as intracanal medicament. Oral Surg. Oral Med. Oral Pathol. Oral Radiol. Endod..

[37] Gama T.G., de Oliveira J.C.M., Abad E.C., Rôças I.N., Siqueira J.F. (2008). Postoperative pain following the use of two different intracanal medications. Clin. Oral Investig..

[38] Barbour M.E., Maddocks S.E., Wood N.J., Collins A.M. (2013). Synthesis, characterization, and efficacy of antimicrobial chlorhexidine hexametaphosphate nanoparticles for applications in biomedical materials and consumer products. Int. J. Nanomed..

[39] Wood N.J., Maddocks S.E., Grady H.J., Collins A.M., Barbour M.E. (2014). Functionalization of ethylene vinyl acetate with antimicrobial chlorhexidine hexametaphosphate nanoparticles. Int. J. Nanomed..

[40] Yue I.C., Poff J., Cortés M.E., Sinisterra R.D., Faris C.B., Hildgen P., Langer R., Shastri V.P. (2004). A novel polymeric chlorhexidine delivery device for the treatment of periodontal disease. Biomaterials.

[41] Blasi P., D’Souza S.S., Selmin F., DeLuca P.P. (2005). Plasticizing effect of water on poly(lactide-co-glycolide). J. Control. Release.

[42] Burdick J.A., Anseth K.S. (2002). Photoencapsulation of osteoblasts in injectable RGD-modified PEG hydrogels for bone tissue engineering. Biomaterials.

[43] Margelos J., Eliades G., Verdelis C., Palaghias G. (1997). Interaction of calcium hydroxide with zinc oxide-eugenol type sealers: A potential clinical problem. J. Endod..

[44] Singh R.P., Ramarao P. (2013). Accumulated polymer degradation products as effector molecules in cytotoxicity of polymeric nanoparticles. Toxicol. Sci..

[45] Cabiscol Català E., Tamarit Sumalla J., Ros Salvador J. (2000). Oxidative stress in bacteria and protein damage by reactive oxygen species. Int. Microbiol..

[46] Singh R.P., Ramarao P. (2012). Cellular uptake, intracellular trafficking and cytotoxicity of silver nanoparticles. Toxicol. Lett..

